# Non-ablative 1927 nm Fractional Thulium Laser With Bimatoprost to Treat a Hypopigmented Burn Scar

**DOI:** 10.7759/cureus.89116

**Published:** 2025-07-31

**Authors:** Connor Latterman, Joyce I Imahiyerobo

**Affiliations:** 1 Dermatology, Vibrant Dermatology, Dedham, USA

**Keywords:** hypopigmentation, laser burn, thulium laser, topical bimatoprost, topical tacrolimus

## Abstract

Hypopigmented scars present a cosmetic and psychological burden, especially in patients of darker skin types. Novel strategies to restore pigment are limited and often ineffective.

A 48-year-old woman presented with a complaint of hypopigmented burn scars on both arms. Two months prior, the patient received an intense pulsed light (IPL) treatment at a medical spa. At her initial consultation, she was prescribed Ruxolitinib (a Janus Kinase (JAK) inhibitor) 1.5% (Opzelura) cream to use each morning and tacrolimus 0.1% ointment (a calcineurin inhibitor) to use every night. After six weeks of applying topicals, she then received two non-ablative 1927 nm fractional thulium laser treatments with bimatoprost 0.03% ophthalmic solution (Latisse) six weeks apart while continuing her topical routine at home. After 18 weeks of treatment, most of the patient’s pigment had returned.

The laser created micro-channels for enhanced topical absorption, while the topical agents reduced inflammation and promoted melanogenesis. This combination offers a promising, non-invasive treatment for hypopigmented burn scars.

## Introduction

Hypopigmented scars are a common result of burns from cosmetic procedures such as intense pulsed light (IPL) Therapy, which can be particularly distressing, with few viable treatment options. In severe cases, melanocyte damage may occur, leading to permanent loss of pigmentation, which can have significant psychosocial effects. Topical treatment alone often yields suboptimal results, especially in patients with Fitzpatrick Skin Types III-IV. A previous study details the use of fractionated carbon dioxide laser with topical latanoprost to treat hypopigmented burn scars; however, side effects included pain, bleeding, pruritus, burning, and contact dermatitis, with 11/14 of the patients recording 25-75% improvement [1]. Previous studies have used microneedling with bimatoprost 0.03% ophthalmic solution (Latisse) to treat hypopigmented scars, but there are no documented reports using a non-ablative 1927 nm fractional thulium laser [2]. This laser, combined with Latisse, has the potential to fully re-pigment without the risk of downtime and side effects from a fractionated carbon dioxide laser.

IPL therapy, widely used to treat hyperpigmentation and sunspots, is a light-based treatment. It works by emitting polychromatic light within a wavelength of 400-1400 nm, which is then absorbed by chromophore molecules in the skin and converted into thermal energy, damaging the target area and thus reducing pigment [3-5]. IPL can overheat the epidermis in patients with darker skin, containing more melanin, increasing cytokines and inflammatory markers. This inhibits melanogenesis and the transfer of melanin to keratinocytes. In severe burns including the dermis, melanocyte apoptosis can cause irreversible hypopigmentation [6].

To address this, the non-ablative 1927 nm fractional thulium laser has emerged as a safe and effective treatment modality for re-pigmentation in hypopigmented scars. It is commonly used in cosmetic dermatology to treat melasma, sun damage, uneven skin texture, hyperpigmentation, and androgenic alopecia. It is safe to use on all Fitzpatrick skin types. The laser works by thermally creating micro-channels in the skin while keeping the stratum corneum intact [7,8]. The micro-channels stimulate collagen production and allow for drug delivery into deep layers of the skin. Latisse, a F2α synthetic prostaglandin analog most used to treat eyelash hypotrichosis and open-angle glaucoma, has been used off-label to stimulate melanogenesis through tyrosinase activation and melanocyte dendricity [7,9,10].

For optimal results, at-home topical therapy is used alongside laser treatments. Tacrolimus 0.1% is a topical calcineurin inhibitor that reduces proinflammatory cytokines like interferon-gamma (IFN-γ) and drastically increases the proliferation of melanin production by increasing tyrosinase activity [11-14]. Ruxolitinib (a Janus Kinase (JAK) inhibitor) 1.5% (Opzelura) cream blocks the JAK-STAT pathway, preventing IFN-γ-mediated melanocytic destruction [15].

## Case presentation

**Figure 1:**
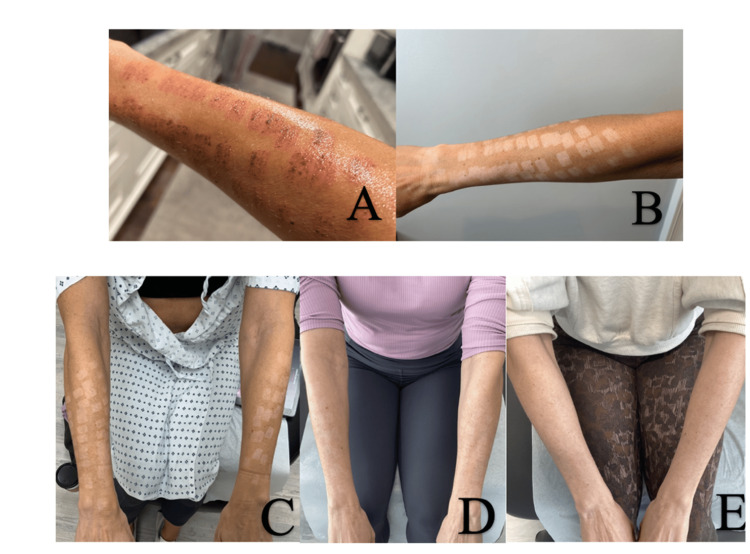
A) Burn scar from intense pulsed light (IPL). B. Healed IPL burn scar with hypopigmentation. C. Hypopigmented scar prior to treatment. D. After six weeks of daily tacrolimus 1% ointment and Opzelura 1.5% cream. E. Six weeks post-laser with Latisse 0.3%, plus daily tacrolimus 0.1% ointment and Opzelura 1.5% cream showing re-pigmentation.

A 48-year-old woman of Southern Italian descent (Type III skin type) presented with a complaint of hypopigmented burn scars on both arms (Figure 1). Physical examination showed hypopigmented squares distributed across both distal arms. The patient was burned on both arms two months prior at a med spa after receiving an IPL treatment. The hypopigmented burn scars did not improve on their own with time or with the use of a topical steroid prescribed by another dermatologist. The patient reported that the burns greatly impacted her self-confidence. To decrease inflammation and return pigment, she was prescribed Opzelura to use each morning and tacrolimus 0.1% ointment to use every night. At her six-week follow-up, partial re-pigmentation was noted in the affected areas.

**Figure 2:**
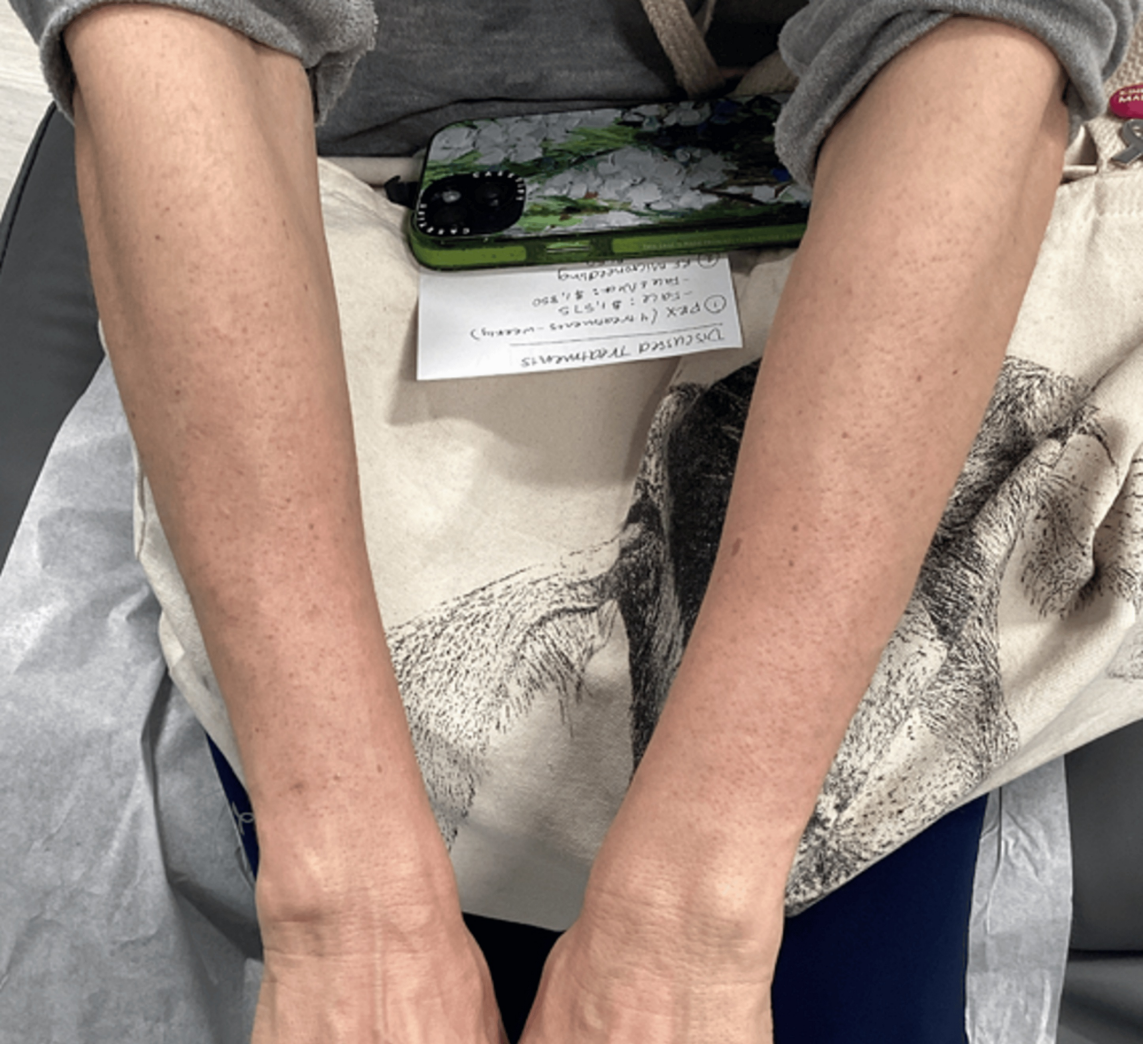
Four weeks post-treatment number 2 with Latisse 0.3% while using tacrolimus 0.1% ointment and Opzelura 1.5% cream daily at home, the pigment had returned to baseline.

Non-ablative 1927nm fractional thulium-doped fiber laser treatments with bimatoprost ophthalmic solution 0.03% were started and repeated every six weeks. It is a quasi-continuous laser generating 40 pulses per shot in an approximate area of 10 mm × 40 mm and a density of 100 mB/cm2. The laser creates micro-channels to a depth of 200 µm in the skin, penetrating much deeper than the depth of melanocytes (35-40 µm) [16,17]. For the first procedure, the laser device was applied to the skin with a power of 16 W and an energy of 12 mJ for five passes. Latisse was then gently massaged into the skin using a cotton swab and allowed to penetrate deep into the skin through the micro-channels created by the laser. Then, her arms were wrapped with wet gauze, and she was instructed to leave it on overnight. The Latisse was not applied at home, only post-procedure. Six weeks post-treatment, a drastic increase in skin pigment was noted in the hypopigmented squares (Figure 1). At this visit, a second laser treatment was conducted with a power of 16W and an energy of 10mJ for five passes. At her follow-up visit post-treatment number two, all pigment was returned to her arms (Figure 2).

## Discussion

This case report details the effectiveness of a three-step, minimally invasive protocol to treat hypopigmented burn scars: fractional laser-assisted drug delivery, prostaglandin analog therapy, and immune-modulating topicals.

The 1927 nm fractional thulium laser initiated dermal remodeling and created micro-channels for drug delivery of Latisse into the dermis. This laser was used instead of other modalities like microneedling to also treat residual hyperpigmentation around the hypopigmented burn scars to blend and create a more uniform skin tone, offering a more cosmetically pleasing result. Microneedling would have provided a method for drug delivery, but it lacks the ability to target pigmentation. Due to significant re-pigmentation following the first treatment, the laser energy was reduced for the second procedure to prevent over-hyperpigmentation.

Latisse is an F2α synthetic prostaglandin analog most used to treat eyelash hypotrichosis and open-angle glaucoma. Many patients using this medication reported eyelid hyperpigmentation as an adverse side effect [9]. Human skin melanocytes contain prostaglandin (PGF) receptors on their surface. These receptors are the main target of prostaglandin F2α (PGF2α) and its analogues [10]. PGF2α stimulates expression of tyrosinase, the rate-limiting enzyme in melanin synthesis, and activates melanocyte dendricity [9]. Therefore, hyperpigmentation may occur due to topical prostaglandin use on the skin. Capitalizing on the re-pigmenting capabilities of Latisse, it has been used off-label in the treatment of vitiligo, an autoimmune disorder characterized by circumscribed white patches of depigmentation due to an acquired lack of functional melanocytes [9,18].

Opzelura is a topical JAK inhibitor that disrupts the JAK-STAT signaling pathway, inhibiting immune-mediated inflammation. The JAK-STAT pathway aids IFN-γ signal transduction, upregulating gene transcription of inflammatory cytokines and chemokines, leading to melanocyte destruction [15]. In July 2022, it was approved for the treatment of non-segmental vitiligo in children aged 12 years and older [18]. By blocking the JAK-STAT pathway, IFN-γ is unable to modulate gene expression and induce melanocyte apoptosis. Like vitiligo, burns also damage melanocytes and release IFN-γ as part of the inflammatory healing process. Hence, Opzelura may be used off-label to treat non-vitiligo hypopigmentation.

## Conclusions

This case report presents a novel and effective treatment using a non-ablative 1927 nm fractional thulium laser administered with bimatoprost and topical immunomodulators (tacrolimus and Opzelura) to re-pigment a burn scar. Often, hypopigmented scars are resistant to treatment and cause significant cosmetic and psychological distress. Clinically, this approach offers a promising therapeutic option for patients with challenging hypopigmented scars, especially patients of darker skin types, where re-pigmentation is often difficult. This case report underscores the potential for integrating laser and topical therapies to achieve a cosmetically pleasing result in re-pigmenting burn scars, providing a reproducible treatment method for dermatologists managing similar hypopigmented scars.
